# Research in orthopedics: A necessity

**DOI:** 10.4103/0019-5413.55968

**Published:** 2009

**Authors:** Anil K Jain

**Affiliations:** *Editor, Indian Journal of Orthopedics, University College of Medical Sciences, University of Delhi, Delhi-110 095, India*

Orthopedics had become a separate specialty within whole field of medicine about 50 years ago.^1^ The Indian Orthopedic Association was a section of Association of Surgeons of India till 1986 before it became an independent society. Orthopedic departments in general were a division of the general surgery till 1970 in India.

The demands of the orthopedist have increased tremendously during the last 30 years because of changes in the demographic profile of population, increase in life expectancy, rapid urbanization and increasing speed of mobility. In developed countries the burden of orthopedic disease is because of aging population and social emphasis on the welfare of elderly. In low income countries it is because of increasing population, rapid urbanization and using high speed vehicles with poor road safety and driving standards. The abysmal health and living standards continue propagation of infective conditions. The increased life expectancy has added aging population.

The advances in medicine have largely been empirical. The intuition has guided development through trial and error. Science has explained what was observed by clinicians.^2^ Now orthopedics has evolved into an advanced specialty in view of research in biomechanics, biomaterials, electrophysiology, molecular biology and genetics and advances in anesthesia, critical care and surgical practices.^1^ The understanding of the human genome, use of genes, the delivery of growth factors and biologically active agents may enhance healing of fractures and disease and help in stopping the progression or even halt developmental and genetic disease. The rapidity and nature of recent scientific developments have provided the potential for an astonishing array of novel advances that will suppress the old treatment.^2^ It seems that more interventions will come from laboratory than from clinical experience.^2^

Most of the orthopedic surgeons repair deficiencies in musculoskeletal system by established surgical methods^1^ while only a few are actively involved in the development and testing of new technology and scientific research. Very little is known about epidemiology, etiology and prevention of musculoskeletal diseases in orthopedics. There is a worldwide decline in the number of orthopedic surgeons involved in research and acting as principal investigator for various projects and the orthopedic surgery resident pursuing education in basic research.^2^

Orthopedic practices till now were guided by the research conducted in the West since the disease profile was more or less the same. In the present times the disparity between disease profiles has widened. The affluent nations have better living standards and health infrastructure. As a result the infective diseases are practically eliminated and degenerative problems are in plenty. Most of the research direction and funding is towards their needs while the low income countries which have 2/3 of world population still see the natural history of disease. They see the continued pathogenesis of osteoarticular infection.^4^ The health infrastructure is over burdened with patients and the clinicians are occupied with patient care; hence they are able to provide basic health support.^3^ We still get simplest of clinical conditions to most challenging cases. We do get a large number of fresh fractures but late presentation of fractures, ununited fractures with broken implants and infected nonunions are also very commonly seen. There may be a locally relevant disease and treatment solutions not be applicable to the western world.^4^ Developed nations continue to research on the clinical problems they encounter. They have no reason to spend funding on clinical problems which they do not face. Since the world is facing a dissimilar clinical disease profile now, a solution has to be region-specific. There is an urgent need for low income countries to conduct research on their clinical problems.

The research includes a clinical research and basic science research. Not many clinical problems can be solved by clinical research alone.^1^ The information about the underlying biological process is provided by the input of basic sciences. There has to be a balance of basic science information to be used by clinicians. The problem of basic and clinical scientists is that they concentrate on the methods which they know well rather than on the question they are trying to answer. Instead of fixing the question to the answer we need to find an answer to a given question. Any progress in the field of Medicine results from original basic and clinical research. Basic research gives us a new and deeper understanding of health, disease and healing.^4^ Clinical research guides us as to how to improve the diagnosis and treatment and provide leads to new direction in basic research.^5^ Development and evaluation of new technologies ultimately requires both basic and clinical research. We know that stem cell, given a different stimulus and milieu, can transform to bone, cartilage, muscle and many other cell lineage. However, we need to define the triggers as well as mediators to these triggers and continuation of the process. Unfortunately most of the development has taken place on implant and biomaterials and little at the cellular level.

We must resolve that basic and clinical research are two sides of one coin; both inseparable. Both require similar commitment and both must be integrated in the same clinical setting. Basic research is largely being conducted by nonphysicians world wide with a gradual decline in orthopedic surgeons as researchers. Basic scientists bring skill to the research proposal but find it difficult to appreciate the clinical relevance. Sarmiento believed, and rightly so, that an orthopedic surgeon who is trained as a clinician-scientist and who understands the capabilities and limitations of the basic science approaches is a superior collaborator.^6^ It would be in the best interest of the speciality if this research is conducted by orthopedic surgeons trained as clinicians and researchers or in a laboratory/departments where orthopedic surgeons and nonphysicians are a team.

The number of clinician scientists has taken a steep decline in the last 30 years in America and Europe in spite of the state of art research facilities and adequate funding.^2^ Jackson DW has listed the common deterrents to young orthopedic surgeons becoming clinician scientists as lack of role model, lack of infrastructure, lack of definition, scope and perceived security in this career path, financial burden (education debt), family responsibility and pressure to earn more, pressure of patient care by institution, lack of financial support for additional training in research, peer pressure to earn fame by successful clinical practice.^7^ There is a wrong notion that basic research is to be conducted by a PhD scientist and orthopedic surgeons can't play a role in this.

On the contrary, low income countries have not yet started thinking about region specific basic research. They have very little infrastructure for research. The attitude of health planners is to support patient care. The education and research is left to the interest of orthopedic clinicians. Hence all research in these countries is conducted by clinicians because of their love towards research. The end result is that there is a very little contribution from these countries to the pool of knowledge. Even society does not recognize the researchers, while clinicians are recognized well. The low income countries should devote resources and part of health budget towards need based research. This has to be realized by teachers, orthopedic associations (whether of countries or region) and government health planners.^5^ We need to bring a change in attitude. The research in these countries should not be done on heterogeneous subjects. Such research may add to the scientific pool of knowledge but is not problem solving. It may be worthwhile to conduct a study to document observations on one research question but, on a similar subject, a series of research questions will give a summative solution to the problem. We need to pick certain disease areas and have an all out approach to create credible research and solution to burning problem. This may include research on etiology, epidemiology and prevention and treatment of musculoskeletal disease. The low income countries have scarcity of funds to develop infrastructure, hence centralized facility should be created for research to be used by one and all. The government should consider research and teaching as equally important components of patient care. A separate time allocation during the week should be given for research activities. Future progress in career should be linked to academic performance. The credible research done rather than the number of publications should be the criteria for promotions and rewards.

Training programs on research methodology should be arranged. We should equip young clinicians with the skills to understand and appreciate the musculoskeletal biology and perhaps conduct serious research on locally relevant issues.^3^ The teaching departments should hold journal clubs and seminars to sensitize students to conduct basic or clinical research. There has to be a group that includes scientists for the study of biological process as well clinician scientists on the project of clinical relevance. The associations should facilitate the process by providing start up research fund. We need a cadre of orthopedic clinician scientists involved in basic, disease oriented, patient oriented and prevention oriented research.^7^

Low income countries have high income countries as an example. The progress in the field of research in orthopedics, the obstacles faced and solutions suggested by them are available. We should ensure credible research programs keeping all these lacunae and matching limited research facilities and heavy disease burden in mind. We should attract the best students in orthopedics for research by providing a structured research curriculum, appropriate funding and reward good outcome and support financial loss which they occurred by not practicing orthopedic surgery.^8^

Indian Journal of Orthopaedics is making a step in this direction. We have three invited write ups in the Perspectives section from well established clinician-scientists from USA, South Africa and India to give their opinion on the (a) need and scope of basic research (b) prospects of basic research in their countries and (c) infrastructure needed for basic research. This is an attempt to sensitize the orthopedic community and Government in this direction.
